# Nephroprotective effect of exercise training in cisplatin-induced renal damage in mice: influence of training protocol

**DOI:** 10.1590/1414-431X2022e12116

**Published:** 2022-08-15

**Authors:** A.A. Almeida, T.M.L. Correia, R.A. Pires, D.A. da Silva, R.S. Coqueiro, M. Machado, A.C.M. de Magalhães, R.F. Queiroz, T.J. Soares, R. Pereira

**Affiliations:** 1 Núcelo de Pesquisa em Fisiologia Integrativa, Departamento de Ciências Biológicas, Universidade Estadual do Sudoeste da Bahia, Jequié, BA, Brasil; 2 Programa de Pós-Graduação Multicêntrico em Ciências Fisiológicas (Sociedade Brasileira de Fisiologia), Universidade Federal da Bahia, Vitória da Conquista, BA, Brasil; 3 Programa de Pós-Graduação Multicêntrico em Bioquímica e Biologia Molecular (Sociedade Brasileira de Bioquímica e Biologia Molecular), Universidade Estadual do Sudoeste da Bahia, Vitória da Conquista, BA, Brasil; 4 Programa de Pós-Graduação em Enfermagem e Saúde, Universidade Estadual do Sudoeste da Bahia, Jequié, BA, Brasil; 5 Programa de Pós-Graduação em Biociências, Universidade Federal da Bahia, Campus Anísio Teixeira, Vitória da Conquista, BA, Brasil; 6 Fundação Universitária de Itaperuna, Itaperuna, RJ, Brasil; 7 Laboratório de Fisiologia e Biocinética, Faculdade de Ciências Biológicas e da Saúde, Universidade Iguaçu Campus V, Itaperuna, RJ, Brasil

**Keywords:** Nephrotoxicity, Physical exercise, Nephroprotection, Redox balance, Swimming

## Abstract

Cisplatin is an effective antineoplastic agent, but its use is limited by its nephrotoxicity caused by the oxidative stress in tubular epithelium of nephrons. On the other hand, regular exercise provides beneficial adaptations in different tissues and organs. As with many drugs, dosing is extremely important to get the beneficial effects of exercise. Thus, we aimed to investigate the influence of exercise intensity and frequency on cisplatin-induced (20 mg/kg) renal damage in mice. Forty male Swiss mice were divided into five experimental groups (n=8 per group): 1) sedentary; 2) low-intensity forced swimming, three times per week; 3) high-intensity forced swimming, three times per week; 4) low-intensity forced swimming, five times per week; and 5) high-intensity forced swimming, five times per week. Body composition, renal structure, functional indicators (plasma urea), lipid peroxidation, antioxidant enzyme activity, expression of genes related to antioxidant defense, and inflammatory and apoptotic pathways were evaluated. Comparisons considered exercise intensity and frequency. High lipid peroxidation was observed in the sedentary group compared with trained mice, regardless of exercise intensity and frequency. Groups that trained three times per week showed more benefits, as reduced tubular necrosis, plasma urea, expression of *CASP3* and *Rela* (NFkB subunit-p65) genes, and increased total glutathione peroxidase activity. No significant difference in *Nfe2l2* (Nrf2) gene expression was observed between groups. Eight weeks of regular exercise training promoted nephroprotection against cisplatin-mediated oxidative injury. Exercise frequency was critical for nephroprotection.

## Introduction

Acute kidney injury (AKI) is characterized by reduced renal function (1) and high morbidity and mortality. Cancer patients treated with the antineoplastic agent cisplatin commonly develop AKI (2,3). Although cisplatin effectively treats certain cancer types, its use in clinical practice is limited because it causes oxidative stress in the tubular epithelium of nephrons (3,4), resulting in nephrotoxicity.

Cisplatin-induced redox imbalance triggers acute tubular necrosis in the proximal tubular epithelium with subsequent vascular dysfunction, intense inflammatory response, and apoptosis (3-6). Given that oxidative stress is the mechanism underlying cisplatin-induced nephrotoxicity, several antioxidant agents have been tested as renal protective agents, including many anti-inflammatory and antioxidant agents (7,8).

Regular physical exercise promotes beneficial adaptations for active muscles and different tissues and organs. These adaptations occur at cellular and systemic levels (9). Increased antioxidant enzyme activity in organs, such as brain, liver, heart, and kidneys, has been reported after exercise training (10-12). However, studies regarding the effectiveness of exercise-induced enhancement of antioxidant activity in oxidative stress-induced kidney damage are scarce.

Regular physical exercise is a promising non-pharmacological intervention to improve renal antioxidant defense and attenuate cisplatin-induced nephrotoxicity. Francescato et al. (6) trained rats with cisplatin-induced AKI for four weeks (running on a treadmill, five times per week) with alternating volume and intensity of training sessions (continuous running for 60 min at 50% of maximal lactate steady-state or 30 min at 100% of maximal lactate steady-state). They observed improved renal function and reduced inflammatory response and tubule-interstitial injuries compared with controls.

Exercise volume, intensity, frequency, duration, and type may influence the level of antioxidant adaptations (13,14). However, the impact of different exercise training protocols on kidney damage mediated by oxidative stress remains unclear. Therefore, this study aimed to investigate the nephroprotective effect of exercise training intensity (low or high) and frequency (three or five times per week) for eight weeks on cisplatin-induced kidney damage in mice.

## Material and Methods

### Animals and experimental protocols

Forty 16-week-old male Swiss mice (40.3±0.7 g) were provided by the Animal Breeding Center of Universidade Estadual de Feira de Santana (Brazil). Animals were housed in polycarbonate cages at the Universidade Estadual do Sudoeste da Bahia (Brazil) and maintained under a 12-h light/dark cycle at standard room temperature (23°C). Water and chow were provided *ad libitum*.

All experimental procedures were conducted following the National Institutes of Health (NIH) Guide for the Care and Use of Laboratory Animals and approved by the Animal Experimentation Committee of the Universidade Estadual do Sudoeste da Bahia (protocol No. 125/2016).

Initially, mice were exposed to five days of familiarization to swimming exercise (five min with load of 1% animal body weight [BW]). Animals were placed in an adapted swimming apparatus, as described by Evangelista et al. (15), consisting of a glass tank (35×35×50 cm) and four acrylic bays (35×15×15 cm for each bay). Two days after the familiarization period, animals initiated eight weeks of forced swimming training with different intensity and frequency for each group as described below.

**Figure 1:**
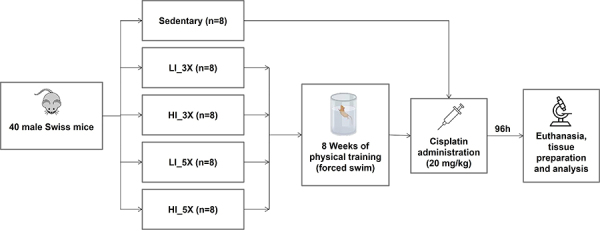
Experimental design. LI: low intensity; HI: high intensity; 3X: three times/week; 5X: five times/week.

Animals were randomly divided into five groups (n=8 animals per group), according to training protocol: 1) sedentary group: no exercise training; 2) LI_3X group: low-intensity training, three times per week for 15 min with load of 2.5% of BW; 3) HI_3X group: high-intensity training, three times per week for 15 min with load of 5% of BW; 4) LI_5X group: low-intensity training, five times per week (Monday to Friday) for 15 min with load of 2.5% of BW; 5) HI_5X group: high-intensity training, five times per week (Monday to Friday) for 15 min with load of 5% of BW. Groups that trained three times per week performed exercises on alternate days (Monday, Wednesday, Friday). A bag with small lead spheres was attached to the tail base of each animal. Workload was adjusted weekly to keep the proposed load constant (i.e., 2.5% of BW for LI_3X and LI_5X; and 5% of BW for HI_3X and HI_5X groups - Figure 1).

BW and naso-anal length were measured weekly during the experiment using a digital scale (VL-3200H, Shimadzu, USA) and non-elastic measuring tape, respectively. The Lee index was calculated based on these measures.

All animals received a single intraperitoneal injection of cisplatin (20 mg/kg; #C2210000, Sigma-Aldrich, USA) two days after the end of the training protocol (1,16) to induce severe kidney damage (17). Mice were anesthetized with intraperitoneal administration of xylazine (16 mg/kg) and ketamine (50 mg/kg) ninety-six hours after cisplatin administration. Blood samples were collected in heparinized tubes by cardiac puncture, and mice were euthanatized by cervical dislocation. This period was chosen because it has been reported as the time required to induce significant kidney damage, especially tubular necrosis (18).

Blood samples were centrifuged (2000 *g* at 4°C for 15 min), and plasma was stored at -70°C for further biochemical analysis. Median laparotomy was conducted for kidney removal. The right kidney was weighed and stored at -70°C for subsequent redox state and gene expression analysis. The left kidney was stored in Metacarn fixative solution (60% methanol, 30% chloroform, and 10% acetic acid) for 24 h and paraffinized for histological analysis. Skin, subcutaneous adipose tissue, internal organs, head, tail, and extremities of anterior and posterior limbs were removed, and bones and muscles were weighed to determine lean body weight (LBW).

### Kidney structure and function

Paraffin-embedded kidney tissue samples were cut into 40-μm sections using a microtome (Leica RM2125RTS, Germany). Sections were deparaffinized with xylene, hydrated with water, and diluted in ethanol before staining with hematoxylin and eosin. Tubular injury was assessed by counting the number of necrotic tubules under light microscopy. Thirty consecutive fields from renal cortex and outer medulla of hematoxylin and eosin-stained sections were photographed at 200× magnification using a camera (Kontron Electronik KS-300, Germany) coupled to a light microscope (BX51, Olympus, Japan).

Renal function was estimated by determining the concentration of urea in plasma using an automated colorimetric enzyme assay kit in an AU680 Chemistry Analyzer (BeckmanCoulter, USA).

### Determination of lipid peroxidation, peroxidase, and total glutathione peroxidase activities

For lipid peroxidation analysis, renal tissue (100 mg/mL) was homogenized in RIPA buffer (Sigma-Aldrich) and centrifuged at 1600 *g* for 10 min at 4°C (Z 36 HK, Hermle-Labortechnik, Germany). Supernatant was collected to determine thiobarbituric acid reactive substances (TBARS), as described by Draper et al. (19). TBARS levels were calculated based on the standard curve of malondialdehyde (MDA) (Cayman Chemical, USA) (0 to 50 µM). TBARS levels (μM) in renal tissue were normalized by protein concentration (mg/mL) and reported as μM/mg protein.

Peroxidase activity was measured by disappearance of H_2_O_2_ at 240 nm (20), while total glutathione peroxidase (GPx) activity was determined according to Paglia and Valentine (21). Peroxidase and GPx activities were normalized by protein concentration (mg/mL) and are reported as mU/mg protein.

Total protein concentration from kidney homogenates was determined by Bradford colorimetric assay at 595 nm (Sigma-Aldrich). Bovine serum albumin (Sigma-Aldrich) (0 to 1.4 mg/mL) was used as standard.

### Quantification of mRNA by real-time qRT-PCR

Total renal RNA was isolated using TRIzol^TM^ reagent (Invitrogen, USA), while the TissueRuptor system (Qiagen, USA) was used for renal tissue homogenization. Complementary cDNA was synthesized from 2 µg of total RNA using a High-Capacity RNA-to-cDNA^TM^ reverse transcription kit (Applied Biosystem, USA).

For quantitative real-time polymerase chain reaction (qPCR), TaqMan^®^ Fast Advanced Master Mix System (Applied Biosystem) was used to examine messenger RNA (mRNA) expression levels of three target genes involved in redox balance, inflammation, and apoptosis, namely *Nfe2l2* (nuclear factor erythroid 2-related factor 2 [Nrf2]; Mm00477784_m1), *Rela* (nuclear factor kappa B [NF-κB] subunit p65, Mm00501346_m1), and *CASP3* (caspase-3; Mm01195085_m1), respectively. Procedures were performed according to manufacturer's instructions, and thermocycling was performed using StepOne Plus thermal cycler (Applied Biosystem). Gene encoding constitutive protein *Gapdh* (glyceraldehyde-3-phosphate dehydrogenase [GAPDH]; Mm99999915_g1) was amplified as housekeeping gene. Gene expression results are reported as relative expression (fold change) and calculated using the comparative method (2^-ΔΔCt^), as proposed by Livak and Schmittgen (22).

### Statistical analysis

Comparisons were performed between groups (sedentary *vs* LI_3X *vs* HI_3X *vs* LI_5X *vs* HI_5X) and with animals grouped by training intensity (sedentary *vs* LI [LI_3X + LI_5X] *vs* HI [HI_3X + HI_5X]) and frequency (sedentary *vs* 3X [LI_3X + HI_3X] *vs* 5X [LI_5X + HI_5X]). Shapiro-Wilk test was used to verify data normality. Comparisons between groups were performed using one-way ANOVA (body composition parameters, percentage of necrotic tubules grouped by week frequency) followed by Tukey's *post hoc* test or Kruskal-Wallis test (gene expression, GPx activity, peroxidase activity, TBARS level, urea, percentage of necrotic tubules grouped by intensity) followed by Dunn's *post hoc* test. Spearman correlation analysis was used to evaluate the relationship between antioxidant activity after training protocol and *Nfe2l2* expression after cisplatin-induced renal damage. Data are reported as means±SE for one-way ANOVA and median (25-75% interquartile range) for Kruskal-Wallis test. Statistical analyses were performed using Graphpad Prism 7.0 software (GraphPad Software, USA). Statistical significance was set at P<0.05.

## Results

### Body composition

BW, naso-anal length, and Lee index were not significantly different between groups (pre- and post-training/control period and 96 h after cisplatin administration; P>0.05) (Table 1). Likewise, relative LBW and kidney weight were not significantly different between protocols (P>0.05) (Table 2).

**Table 1:** Body weight, naso-anal length, and Lee index obtained before and after eight weeks of training.

	Sedentary	LI_3X	HI_3X	LI_5X	HI_5X
Body weight (g)					
Pre-training	41.8±1.7	40.9±0.9	37.7±2.0	40.0±0.8	40.9±1.4
Post-training	43.4±1.4	41.2±0.8	42.7±1.8	43.8±1.1	42.4±2.5
Post-cisplatin administration^a^	36.3±1.7	33.6±0.9	36.3±1.4	34.8±1.0	35.7±0.5
Naso-anal length (cm)					
Pre-training	11.0±0.2	10.9±0.1	10.8±0.2	10.9±0.3	11.0±0.1
Post-training	11.2±0.2	10.9±0.1	11.1±0.2	11.0±0.3	11.0±0.2
Lee index					
Pre-training	0.31±0.00	0.31±0.01	0.32±0.01	0.32±0.01	0.31±0.01
Post-training	0.32±0.00	0.33±0.00	0.31±0.00	0.32±0.01	0.33±0.01

Data are reported as means±SE. P>0.05 (ANOVA). LI_3X: group trained at low-intensity, three times per week; HI_3X: group trained at high-intensity, three times per week; LI_5X: group trained at low-intensity, five times per week; HI_5X: group trained at high-intensity, five times per week. ^a^Body weight was measured 96 h after cisplatin administration.

**Table 2:** Relative lean body weight and kidney weight after 96 h of cisplatin administration.

	Sedentary	LI_3X	HI_3X	LI_5X	HI_5X
Lean body weight (%)^a^	37.0±0.7	37.3±1.4	38.3±0.5	37.3±1.4	39.7±1.2
Kidney (%)^a^	2.5±0.2	2.1±0.1	2.8±0.2	2.8±0.1	2.3±0.3

Data are reported as means±SE. P>0.05 (ANOVA). LI_3X: group trained at low-intensity, three times per week; HI_3X: group trained at high-intensity, three times per week; LI_5X: group trained at low-intensity, five times per week; HI_5X: group trained at high-intensity, five times per week. ^a^Parameters normalized by body weight.

### Structural and functional renal assessment

**Figure 2:**
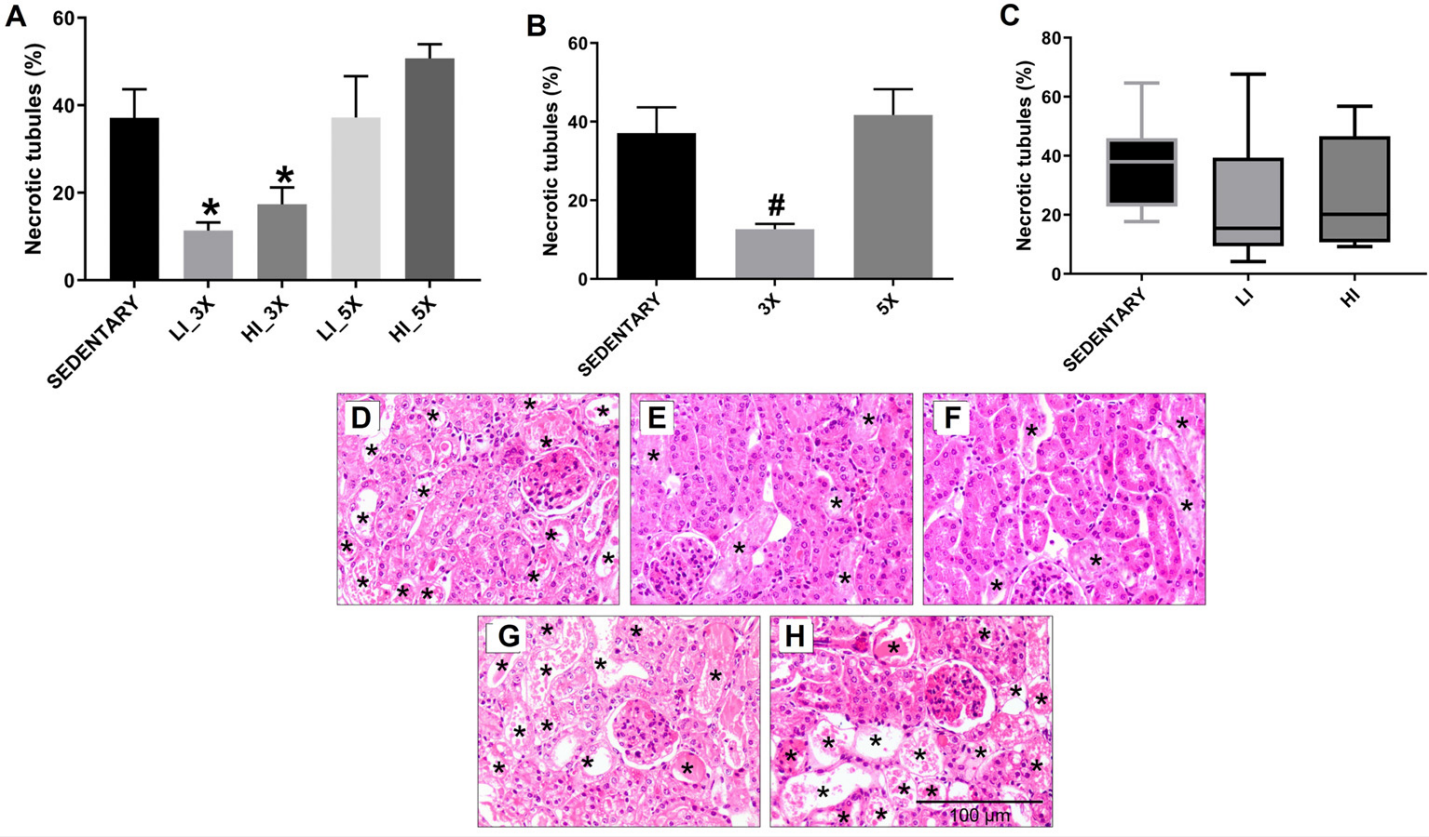
Means±SE of the percentage of necrotic tubules in studied groups (sedentary, trained at low [LI] and high [HI] intensity, three times [3X], and five times [5X] per week) (**A**). Means±SE (**B**) and median and interquartile range (**C**) of the percentage of tubular necrosis of animals grouped by training frequency (3X and 5X per week) (**B**) and training intensity (low and high intensity) (**C**). *P<0.05 compared with LI_5X and HI_5X groups; ^#^P<0.05 compared with sedentary and 5X groups (ANOVA). Representative photomicrographs (200× magnification, scale bar 100 μm) of renal cortex stained with hematoxylin and eosin: (**D**) sedentary group, (**E**) group trained at low intensity, three times per week (LI_3X), (**F**) group trained at high intensity three times per week (HI_3X), (**G**) group trained at low intensity, five times per week (LI_5X), and (**H**) group trained at high intensity five times per week (HI_5X). *Indicates tubular necrosis in panels D-H.

Tubular necrosis was lower in animals trained three times per week (at low and high intensity) than sedentary mice and mice trained five times per week (low and high intensity), as shown in Figure 2A and photomicrographs in Figure 2D-H.

Analysis on training frequency (Figure 2B) and intensity (Figure 2C) revealed that frequency was more determinant for nephroprotection than intensity since mice that trained three times per week demonstrated less tubular necrosis (P<0.05), regardless of intensity. On the other hand, no significant difference was observed between animals grouped by intensity (P>0.05), regardless of frequency.

**Figure 3:**
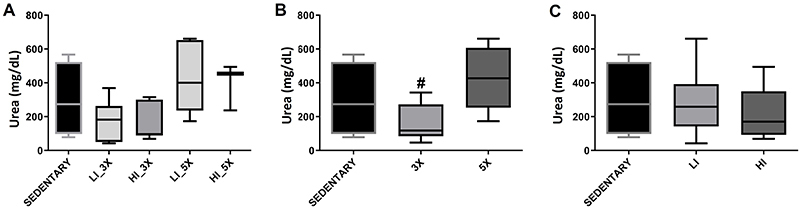
Median and interquartile range of concentration of urea in the plasma of sedentary, trained at low (LI) and high (HI) intensity, three times (3X) and five times (5X) per week groups (**A**) and according to animals grouped by training frequency (three and five times per week) (**B**) and training intensity (low and high intensity) (**C**). ^#^P<0.05 compared with 5X group (Kruskal-Wallis).

Urea concentration in plasma was not significantly different between groups (Figure 3A; P>0.05), except when animals were grouped by training frequency. Mice trained three times per week showed lower urea concentration in plasma than those trained five times per week (Figure 3B; P<0.05), however, there was no difference with training intensities (Figure 3C).

### Levels of lipid peroxidation, peroxidase, and total glutathione peroxidase activity

**Figure 4:**
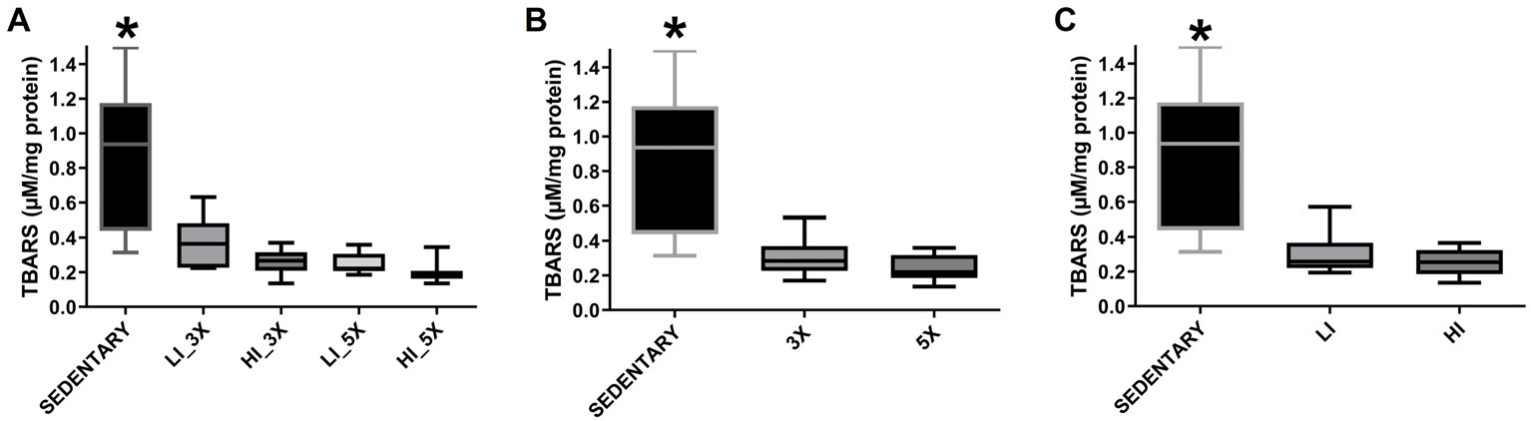
Median and interquartile range of thiobarbituric acid reactive substances (TBARS) level in renal tissue samples from groups (sedentary, trained at low [LI] and high [H]) intensity, three times [3X] and five times [5X] per week) (**A**) and according to animals grouped by training frequency (three and five times per week) (**B**) and training intensity (low and high intensity) (**C**). *P<0.05 compared with all trained groups (Kruskal-Wallis).

TBARS levels in kidney homogenates were significantly higher in the sedentary group than all trained groups (Figure 4A; P<0.05). Lower TBARS production was observed in kidney tissue samples from trained animals, regardless of training frequency (Figure 4B) or intensity (Figure 4C; P<0.05).

**Figure 5:**
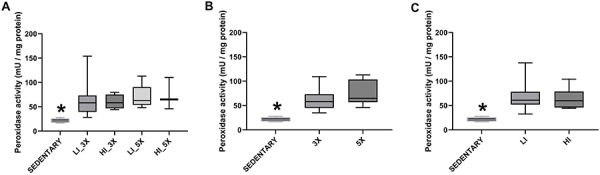
Median and interquartile range of peroxidase activity in renal tissue samples from groups (sedentary, trained at low [LI] and high [HI] intensity, three times [3X] and five times [5X] per week) (**A**) and according to animals grouped by training frequency (three and five times per week) (**B**) and training intensity (low and high intensity) (**C**). *P<0.05 compared with all trained groups (Kruskal-Wallis).

Peroxidase activity was significantly higher in homogenates of all trained groups than of the sedentary group (Figure 5A; P<0.05). Both training frequency and intensity positively influenced peroxidase activity since they exhibited significantly higher catalase activity than the sedentary group (Figure 5B and C; P<0.05).

**Figure 6:**
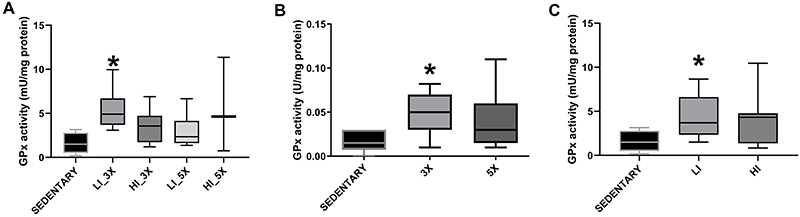
Median and interquartile range of glutathione peroxidase (GPx) activity in renal tissue samples from groups (sedentary, trained at low [LI] and high [HI] intensity, three times [3X] and five times [5X] per week) (**A**) and according to animals grouped by training frequency (three and five times per week) (**B**) and training intensity (low and high intensity) (**C**). *P<0.05 compared with sedentary group (Kruskal-Wallis).

Total GPx activity was significantly higher in renal tissue samples from LI_3X group than the sedentary group (P<0.05; Figure 6A). When grouped by training frequency, mice trained three times per week showed significantly higher GPx activity than the sedentary group (P<0.05; Figure 6B). Likewise, when grouped by intensity, animals trained at low intensity showed significantly higher GPx activity than the sedentary group (P<0.05; Figure 6C).

### Gene expression in renal tissue samples

**Figure 7:**
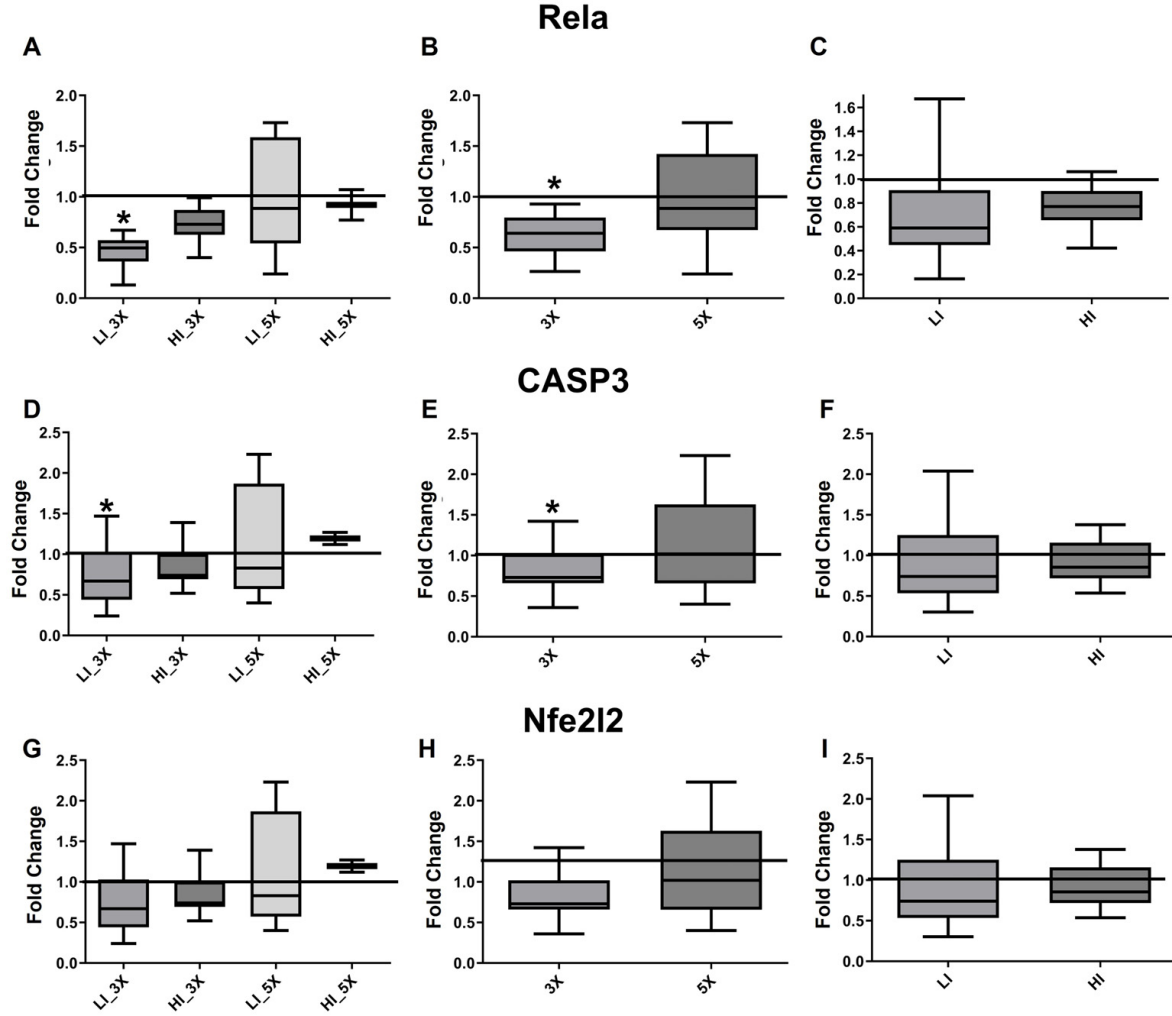
Median and interquartile range of gene expression of *Rela* (NFκB subunit p65) (**A**-**C**), *CASP3* (caspase-3) (**D**-**F**), and *Nfe2l2* (Nrf2) (**G**-**I**) from renal tissue samples after cisplatin-induced renal damage from groups (sedentary, trained at low [LI] and high [HI] intensity, three times [3X] and five times [5X] per week). *P<0.05 compared with sedentary group (Kruskal-Wallis).

*Rela* and *CASP3* gene expression demonstrated similar responses against kidney damage and were significantly lower in kidney tissue samples from LI_3X group than the sedentary group (Figure 7A and D; P*<*0.05). When grouped by frequency, *Rela* and *CASP3* gene expression were significantly lower in groups trained three times per week (Figure 7B and E; P*<*0.05). No significant differences were observed when animals were grouped by intensity (Figure 7C and F; P>0.05).

*Nfe2l2* expression in kidney tissue samples was not significantly different between groups (Figure 7G; P>0.05). Similarly, no significant difference was observed when animals were grouped by frequency (Figure 7H; P>0.05) or intensity (Figure 7I; P>0.05). A negative and significant correlation was found between GPx activity and *Nfe2l2* expression (Spearman correlation coefficient (rho)=-0.72; P*<*0.001), whereas a negative, but not significant correlation, was found between peroxidase activity and *Nfe2l2* expression (correlation coefficient=-0.12; P*=*0.596).

## Discussion

This study investigated the nephroprotective effect of eight weeks of exercise training with different intensities (low or high) and frequencies (three or five times per week) on cisplatin-induced AKI in mice. Our results demonstrated that eight weeks of exercise training induced: 1) low levels of tubular necrosis and urea concentration in plasma of animals trained three times per week, regardless of intensity; 2) low TBARS levels in kidneys of all trained animals; 3) high peroxidase activity in kidneys of all trained animals, and high total GPx activity in groups trained three times per week at low intensity; and 4) low *Rela* (NFκB subunit p65) and *CASP3* gene expression in kidney tissue samples of animals trained three times per week.

### Oxidative-induced kidney damage and antioxidant defense

Increased activity of antioxidant enzymes in several organs has been reported after a period of eight weeks of exercise training (23). The role of this adaptive antioxidant defense against oxidative insult was evaluated in heart and brain (11). Although some studies investigated the protective effects of exercise against kidney dysfunction (12,24,25), the influence of training with different intensities and frequencies remained unclear.

Saad (12) evaluated the protective effect of exercise against oxidative damage due to ischemia or reperfusion in rat kidney and observed that animals trained for 11 weeks (60 min of forced swimming, three times per week) demonstrated low levels of plasma creatinine, urea, TNF-α, and lipid peroxidation in homogenates, with no difference in peroxidase activity. In the present study, we demonstrated a protective effect of exercise in acute and predominant oxidative kidney injury. In line with Saad (12), our results suggested that an interaction between training intensity and frequency may influence potential gains induced by regular exercise.

Miyagi et al. (24) and Estrela et al. (25) investigated the protective effects of exercise training against cisplatin-induced renal injury in mice. Similar to the present study, both studies (24,25) administered 20 mg/kg of cisplatin intraperitoneally. Their results confirmed an exercise-induced nephroprotective effect against oxidative insult since the trained group exhibited low levels of tubular necrosis. Miyagi et al. (24) observed improved antioxidant defense by increasing the expression of heme oxygenase 1 (HO-1). Although Estrela et al. (25) did not analyze direct markers of antioxidant defense, inflammatory patterns attenuated in renal tissue of trained animals.

Estrela et al. concluded that a four-week caloric restriction period was more effective than exercise on nephroprotection against cisplatin-induced renal damage (25). Miyagi et al. (24) and our study used a prolonged training period (seven and eight weeks, respectively), allowing us to believe that prolonged periods of exercise training (i.e., longer than four weeks) must be needed to achieve a significant nephroprotection effect. Further studies should identify the minimum period needed for exercise-induced nephroprotective effect.

Miyagi et al. (24) applied a treadmill running exercise protocol lasting 30 to 60 min, five times per week for seven weeks, whereas Estrela et al. (25) used a forced swimming exercise protocol without additional load (60 min, five times per week during four weeks). Although the exact frequency of weekly training remains unclear, our results indicated that this parameter can be crucial to induce adaptation and consequent nephroprotection.

The term “preconditioning” has been used for exercise-induced improvement in tissue protection against oxidative stress (10,23). Although well documented, the benefits of preconditioning depend on the exercise protocol (26). In our study, training frequency influenced exercise-induced adaptations since groups trained three times per week showed better nephroprotection against cisplatin-induced oxidative damage than groups trained five times per week.

Cisplatin-induced kidney damage is primarily caused by oxidative damage in epithelial cells from the proximal tubule, and H_2_O_2_ is recognized as the key oxidant related to cellular death (27,28). Thus, increased peroxidase activity observed in trained animals should explain the reduced level of tubular necrosis. Our findings support the findings of Tsutsumishita et al. (27), Baek et al. (28), and Ma et al. (29) who demonstrated that catalase, an important peroxidase, can attenuate cisplatin-induced nephrotoxic effect. Increased peroxidase activity observed in the present study can be interpreted as an exercise-induced protective effect since the increased synthesis of antioxidant enzymes (e.g., peroxidases and HO-1) is observed after exercise training periods with different intensities and durations (24). However, it is worth noting that, although we observed increased peroxidase activity in all trained groups, only those that trained three times per week, regardless of intensity, showed lower tubular necrosis.

Interestingly, tubular necrosis was inversely related to total GPx activity, especially in animals trained three times per week. Our results suggested that GPx enzyme should contribute to nephroprotection in cisplatin-induced oxidative renal damage after exercise. Also, this difference in GPx activity among animals trained with different frequencies and intensities may indicate an exercise-specific adaptation of this enzyme. Renal epithelium is one of the tissues with higher GPx levels in its classic/cytosolic isoforms (GPx-1) and is responsible for plasma isoforms (GPx-3 and GPx-4). These isoforms, also called phospholipid hydroperoxide GPx (PHGPx) (30,31), act simultaneously to consume hydroperoxides (30).

The relationship between increased nephroprotection in groups trained three times per week, especially with low intensity, and increased total GPx activity could be explained by kinetic characteristics of GPx enzyme. GPx enzyme has a relatively low *Km* value for H_2_O_2_, suggesting effective removal of H_2_O_2_ at low substrate concentrations (32). Thus, exposure to relatively lower H_2_O_2_ levels in low-intensity training exercise three times per week may have facilitated an adaptive response by increasing GPx enzyme levels.

Margonis et al. (32) observed specific GPx activity in blood samples from 12 healthy volunteers submitted to a training program divided into five three-week blocks, in which training volume increased from the first to third block and reduced from the third to fourth block. GPx activity was significantly higher in the transition from second to third training block (i.e., before load increase from moderate to high and training frequency from four to six times per week). However, Margonis et al. (32) and our study present important methodological differences, such as enzyme activity measured in different samples (blood *vs* renal tissue) and species (humans *vs* mice). Considering that GPx enzyme kinetics favor H_2_O_2_ removal at relatively lower concentrations, groups trained with lower volume or prolonged intervals between exercise sessions may facilitate greater GPx activity.

### Gene expression after cisplatin-induced oxidative renal damage

In the present study, *Rela* gene expression was significantly lower in the group trained three times per week, especially at low intensity, than sedentary group. NFkB is a transcription factor that regulates gene expression from a wide range of proteins involved in the inflammatory response, while one of the main targets of signaling cascade is initiated by the TNF-α receptor, a key event in cisplatin-induced renal injury (17). However, NFkB activation is influenced by H_2_O_2_ levels due to increased degradation of the NFkB inhibitory subunit, called IkB (33).

Therefore, increased peroxidase activity, such as peroxiredoxins and GPx, can attenuate pro-inflammatory signaling mediated by TNF-α, justifying the lower expression of *Rela* gene in animals trained three times per week. Moreover, Miyagi et al. (24) and Estrela et al. (25) observed downregulation of *TNF-α* gene expression and TNFR2 in renal tissue of animals submitted to exercise training before cisplatin-induced renal damage. Leite et al. (34) also found a positive effect of an eight-week physical training program, attenuating gene and protein expression of NF-κB/RelA (p65) after cisplatin-induced renal damage. These findings, combined with our results, confirmed a low inflammatory profile for trained animals exposed to oxidative damage in kidneys, which seems to enhance antioxidant defense.

*CASP3* gene expression observed in trained groups can also be related to improved antioxidant defense since attenuation of oxidative damage can reduce cellular death. Higuchi et al. (35) demonstrated that *CASP3* is involved in apoptosis and necrosis processes; the outcome (apoptosis or necrosis) induced by *CASP3* is directly influenced by oxidative stress. Low oxidative stress conditions lead to apoptosis, whereas high oxidative stress conditions lead to necrosis. Additionally, Wang et al. (36) demonstrated that *CASP3* expression correlates negatively with several peroxidases, such as GPx, which justifies the low structural damage observed in groups trained three times per week.

Nrf2 is reported as the primary regulator of antioxidant defense, and its gene expression increases after exercise (37). Previous studies identified that H_2_O_2_ is an important stimulator of Nrf2 expression that regulates the expression of more than 200 cytoprotective genes, including antioxidant enzymes, such as catalase, peroxiredoxins, and GPxs. Therefore, regular exercise induces an increase in Nrf2 expression and, consequently, increases cellular defense capacity by a transient and moderate increase of H_2_O_2_ levels, explaining the increased activity of peroxidases and GPx in trained animals from our study. Therefore, increased antioxidant defense in the training period, especially against H_2_O_2_ (38), might attenuate the stimulus leading to *Nfe2l2* expression after cisplatin-induced oxidative damage. This hypothesis is reinforced by the negative correlation between antioxidant enzyme activity, especially GPx and *Nfe2l2* expression.

In our study, *Nfe2l2* expression showed the expected tendency of being lower in animals trained before cisplatin-induced oxidative damage; however, no significant difference was identified between groups. Although Miyagi et al. (24) found no significant difference in Nrf2 expression in kidneys of animals trained before cisplatin-induced kidney damage, they reported a significant difference in expression of the antioxidant enzyme HO-1.

Since biological adaptations to exercise occur hours or days after the training session (9), intervals between sessions may be needed for tissue adaptations. Hence, our results add new information to the field. While high-intensity exercise programs with high weekly frequency (i.e., high volume) have been suggested to improve performance (39), our training at low intensity and low weekly frequency (i.e., low volume) effectively potentiated antioxidant defense in kidneys. Indeed, our results demonstrated that training frequency has a major influence on nephroprotection, suggesting that an incomplete recovery between training sessions might have increased oxidative stress, impairing the exercise-induced antioxidant defense enhancement.

While a lifestyle with low physical activity level is reported as a risk factor for nephrotoxicity after cisplatin administration (40), regular exercise training may promote a wide range of benefits. Therefore, the inclusion of an exercise training routine as nephroprotective protocol to minimize cisplatin-induced kidney injury should be considered. Also, exercise-induced nephroprotection is potentially mediated by increased antioxidant defense capacity in renal tissue, suggesting that this non-pharmacological therapeutic approach does not interfere with antineoplastic agent, as observed with the use of cisplatin metabolizing inhibitors (i.e., γ-glutamyltranspeptidase [GGT] inhibitors) (5).

In conclusion, the nephroprotective effect induced by regular exercise can be mediated by increasing antioxidant defense capacity in renal tissue, attenuating inflammatory process and cellular death in renal tubules following cisplatin-induced oxidative kidney damage. Our results demonstrated that training frequency has a major influence on nephroprotection.

Results also support the hypothesis that a more active lifestyle is essential to reduce cisplatin-induced nephrotoxicity due to increased antioxidant defense capacity. Exercises performed three times per week, regardless of intensity, were sufficient to increase antioxidant defense of renal tissue. Future studies must investigate whether our findings can be generalized to humans.
